# IL‐18: A potential inflammation biomarker in Wiskott–Aldrich syndrome

**DOI:** 10.1002/eji.202049024

**Published:** 2021-02-05

**Authors:** Elizabeth Rivers, Ying Hong, Mona Bajaj‐Elliott, Austen Worth, Adrian J. Thrasher

**Affiliations:** ^1^ UCL Great Ormond Street Institute of Child Health London UK; ^2^ Great Ormond Street Hospital for Children NHS Foundation Trust London UK

## Abstract

Analysis of serum cytokine levels in Wiskott–Aldrich syndrome patients pre‐ and post‐ treatment reveals IL‐18 as a stable and reliable marker of inflammation. Definitive stem cell treatment with good myeloid correction correlates with resolution of inflammation and reduction of circulating IL‐18, highlighting the importance of actin cytoskeletal regulation of myeloid cells in control of inflammation.

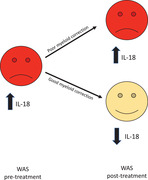

Wiskott–Aldrich syndrome (WAS) is a rare primary immunodeficiency disease caused by dysregulation of the actin cytoskeleton in hematopoietic cells. Clinical features include recurrent infections, thrombocytopenia, and predisposition to hematological malignancy, which necessitate definitive treatment in the form of hematopoietic stem cell transplant (HSCT) or gene therapy. A significant inflammatory phenotype occurs in 70–80% of children, including severe eczema, inflammatory bowel disease (IBD), and vasculitis, which can be difficult to manage [1]. Furthermore, about 20% of patients have ongoing or episodic autoinflammation after HSCT, particularly where myeloid chimerism is low [2, 3, 4, 5]. We sought to characterize the inflammatory signature in WAS patients, with a view to identifying potential biomarkers and opportunities for targeted therapeutic interventions.

Serum cytokine concentrations of IL‐1β, IL‐18, IL‐18bp, IL‐6, TNF‐α, IFN‐γ, and chemokines IP‐10 and MCP‐1 were quantified from healthy donors and WAS patients using ELISA (IL‐18 and IL‐18bp) or multiplex Meso Scale Discovery (MSD) assays. Circulating protein levels were correlated to clinical parameters such as presence of inflammatory symptoms or need for anti‐inflammatory medications and laboratory markers including donor chimerism and vector copy number (VCN) for those post‐HSCT or gene therapy, respectively (Table 1).

**Table 1:** Characteristics of WAS patients in study cohort

Patient	*WAS* mutation	Pre‐/posttreatment (conditioning)	Time post treatment	IL‐18 (pg/mL)	Myeloid correction	Lymphoid correction	Inflammatory features	Anti‐inflammatory medications
1	c.143C>T (p.Thr48Ile)	Post‐HSCT (Flu/Treo/Campath)	8 years	284.3	100%	100%	Resolved eczema	None
2	Deletion of whole gene	Post‐HSCT (Bu/Cyclo)	10 years	211.8	100%	100%	Resolved eczema	None
3	c.374G>A (p.Gly125Glu)	Pre	‐	241.2	‐	‐	Eczema	Eumovate (sirolimus and prednisolone for hemolytic anemia)
3	c.374G>A (p.Gly125Glu)	Post‐HSCT (Flu/Treo/Campath)	1 year	1563.8	44%	98%	Eczema/skin GvHD	Eumovate, prednisolone, sirolimus, infliximab
4	c.302T>C (p.Leu101Pro)	Pre	‐	813.99	‐	‐	Eczema	Eumovate
4	c.302T>C (p.Leu101Pro)	Post‐GT (Bu/Flu)	1 year	179.46	VCN 0.04	VCN 0.02	Eczema	Eumovate (prednisolone for cytopenias)
5	c.1483DelG (p.Asp495Metfs164X)	Pre	‐	804.6	‐	‐	Eczema	Eumovate
5	c.1483DelG (p.Asp495Metfs164X)	Post‐GT (Bu/Flu)	1 year	456.1	VCN 0.18	VCN 0.63	Resolved eczema	None
6	c.250T>C (F84L)	Post‐HSCT (Flu/Melph)	18 years	299.3	0%	91%	Intermittent arthritis, resolved eczema	Naproxen
7	c.250T>C (F84L)	Post‐HSCT (Flu/Melph/Campath)	17 years	127.8	100%	100%	Resolved eczema	None
8	c.97C>T (p.Q33X)	Pre	‐	3000+	‐	‐	Eczema, IBD	Prednisolone, anakinra, topical hydrocortisone
8	c.97C>T (p.Q33X)	Post‐GT (Bu)	1 month	1874.4	VCN 0.92	‐	Eczema, IBD	Prednisolone, anakinra, topical hydrocortisone
8	c.97C>T (p.Q33X)	Post‐GT (Bu)	3 months	297.4	VCN 0.87	VCN 0.38	Improved IBD, resolved eczema	Weaning prednisolone, anakinra
9	c.1225G>T (p.Gly409*) **	Post‐HSCT (Flu/Treo/Campath)	5 years	14459.7	7%	93%	Vasculitis, IBD, eczema	Anakinra, colchicine, sulphasalazine, topical trimovate
10	c.80T>A (p.Leu27His)	Pre	‐	1053.2	‐	‐	Eczema	Eumovate
11	IVS6+2t>c	Post‐HSCT (Flu/Melph/Campath)	15 years	1138.2	0%	85%	Eczema	Eumovate
12	c.913C>T (p.Gln305*) **	Post‐GT (Bu/Flu)	3 years	234.2	VCN 1.43	VCN 1.99	Resolved eczema	Prednisolone and MMF for nephrotic syndrome

Characteristics of WAS patients recorded at the time of sample collection. Myeloid/lymphoid correction refers to percentage of donor chimerism or VCN in CD14^+^ or CD15^+^ cells for patients post‐HSCT or GT respectively.

Bu, busulfan; Cyclo, cyclophosphamide; Flu, fludarabine; GT, gene therapy; GvHD, graft versus host disease; HSCT, hematopoietic stem cell transplant; IBD, inflammatory bowel disease; Melph, melphalan; MMF, mycophenylate mofetil; Treo, treosulfan; VCN, vector copy number; WAS, Wiskott–Aldrich syndrome.

** Patients with additional genetic mutations identified (patient 9: hemizygous mutation in familial Mediterranean fever gene *MEFV* p.726A; patient 12: additional variant in *WAS* gene c.391G>A, p.Glu1331Lys). + cytokine concentration exceeded upper limit of detection.

The study cohort consisted of 12 patients with WAS, from whom samples were collected over a 2‐year period (2017–2019). Four WAS patients were followed through definitive stem cell therapy allowing for serial measurements, giving a total of 16 patient samples, which were compared with 19 healthy control (HC) samples. Of the WAS samples, five were predefinitive treatment and 11 posttreatment with either HSCT or gene therapy. Note that 55% of those post‐HSCT or gene therapy were considered to have partial myeloid correction (<50% donor chimerism or VCN < 0.5 in myeloid cells). All WAS patients prior to definitive therapy were noted to have moderate‐severe eczema. Additional inflammatory features such as IBD, vasculitis, and arthritis were seen in 27% of those either pre‐ or postdefinitive therapy with partial myeloid correction. In contrast, none of the patients at least 1‐year post‐HSCT or gene therapy with good myeloid correction had ongoing inflammatory symptoms (Table 1).

The most striking finding was that serum IL‐18 levels in WAS were significantly elevated when compared with HC, particularly in patients with IBD (Fig. 1A). Interestingly, IL‐18 remained elevated in patients post‐HSCT with partial myeloid correction, but returned to normal in those with good myeloid correction, correlating with the presence or absence of inflammatory symptoms. These observations were further confirmed in a WAS patient in whom multiple sampling was carried out; a decline in circulating IL‐18 level after gene therapy correlated with improvement in inflammatory symptoms and weaning of anti‐inflammatory medications (Fig. 1B and Patient 8, Table 1). Circulating levels of other candidate pro‐inflammatory cytokines and chemokines were otherwise comparable between WAS and healthy donors (Supporting Information Fig. S1).

**Figure 1:**
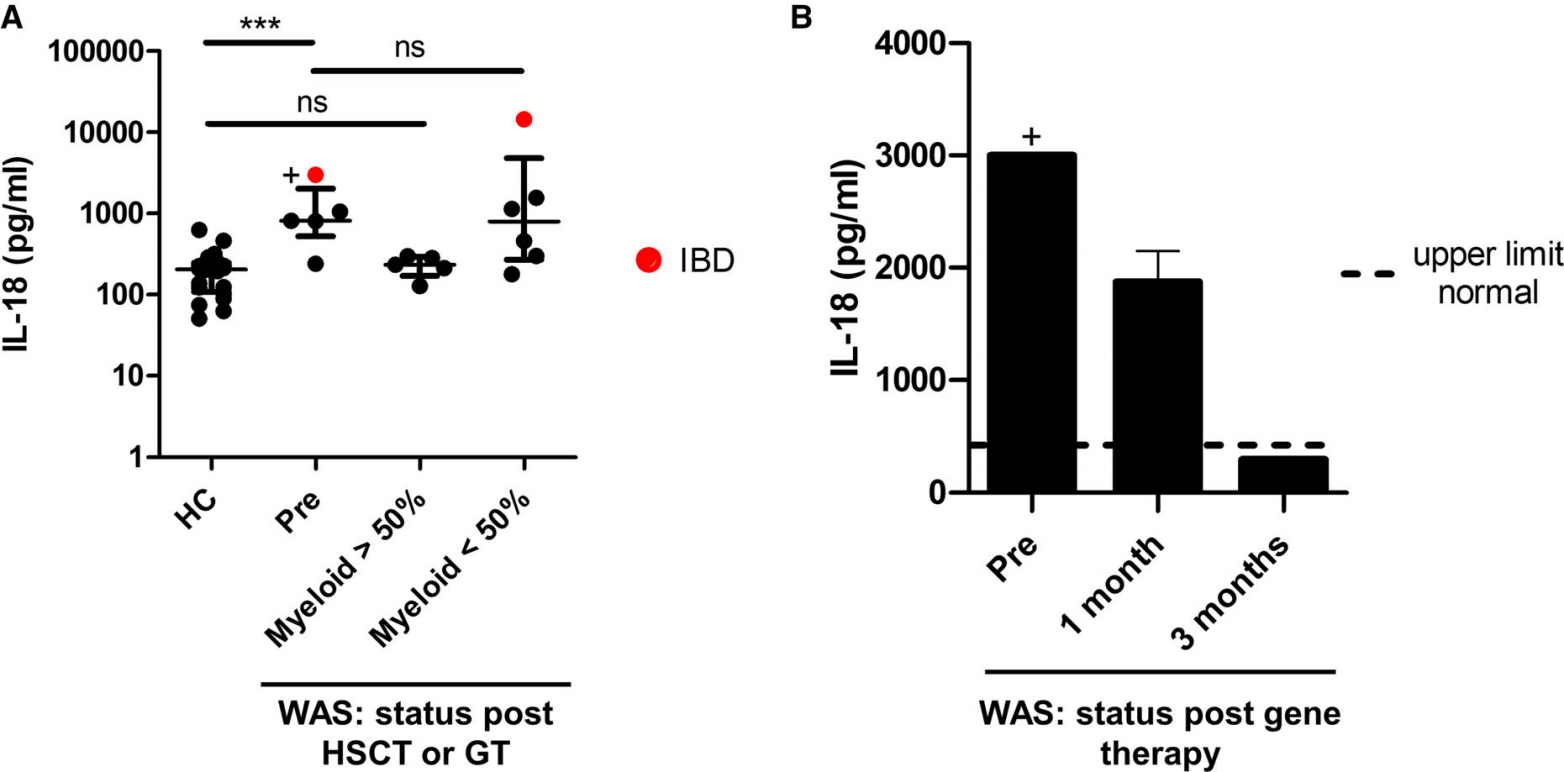
Serum IL‐18 concentration in WAS patients. **(A)** Serum IL‐18 concentration was quantified from 19 healthy donors and 16 WAS patient samples (5 pre‐ and 11 post‐definitive therapy, separated according to myeloid correction), in two separate experiments. Myeloid < or > 50% represents donor chimerism < or > 50% after HSCT or VCN < or > 0.5 after gene therapy. Dots are mean of duplicate values and red dots represent patients with IBD; + cytokine concentration exceeded upper limit of detection, but insufficient sample remained to repeat at greater dilution to determine absolute value. Bars are median ± IQR. Mann–Whitney ***p < 0.0001. **(B)** IL‐18 concentration from the same WAS patient pre‐, 1 month, and 3 months after gene therapy was quantified by ELISA in one experiment. Bars are mean ± SEM for duplicates. Dotted line represents upper limit of normal; + cytokine concentration exceeded upper limit of detection, but insufficient sample remained to repeat at greater dilution to determine absolute value. HC, healthy control; HSCT, haematopoietic stem cell transplant; IBD, inflammatory bowel disease; IQR, interquartile range; ns, not significant; SEM, standard error of mean; VCN, vector copy number; WAS, Wiskott–Aldrich syndrome.

IL‐18 is a member of the IL‐1 family of cytokines. Epithelia and myeloid (monocytes, macrophages, and dendritic cells) lineage are the predominant source of IL‐18. Although originally described as an important inducer of IFN‐γ and Th1 differentiation, increasing evidence supports the expanding role of IL‐18 in mediating inflammation, through activation of Th2, IL‐17‐producing γδ T cells, and macrophages [6]. Release of bioactive IL‐18 is dependent on inflammasome activity [7] and the finding of elevated serum IL‐18 is consistent with our previous findings of dysregulated inflammasome activity in WAS [8]. Bioactive IL‐1β secretion is also inflammasome dependent, and we have previously demonstrated increased IL‐1β secretion in murine and human in vitro models of infection and inflammation [8]. Herein, we did not observe elevated levels of IL‐1β in WAS serum, most likely due to its short half‐life; this labile nature makes it difficult to detect, as reported in patients with other autoinflammatory conditions [9]. Support for a contributing role of IL‐1β in WAS‐associated inflammation, however, is the improvement in symptoms for both of the patients in our cohort receiving the recombinant IL‐1 receptor (IL‐1R) antagonist

Anakinra. This treatment inhibits signaling through the IL‐1R complex, the receptor for IL‐1β and IL‐1α (but not IL‐18). IL‐18 bioactivity is physiologically regulated via its binding protein, IL‐18 binding protein (IL‐18bp). We found no significant difference in serum IL‐18bp levels between WAS patients and HC (data not shown), and the ratio of IL‐18 to IL‐18bp was double that of healthy controls in four patients tested (0.14 vs. 0.076, respectively).

IL‐18 is an unusually stable cytokine due to the lack of gene‐associated RNA destabilizing elements, making it an attractive biomarker of inflammation. The cytokine is increasingly utilized in diagnosis and follow‐up of other autoinflammatory conditions, such as macrophage activation syndrome, systemic juvenile idiopathic arthritis (sJIA), and adult onset Stills disease (AOSD) [6]. We propose that IL‐18 may also be a useful biomarker for inflammation in WAS and speculate that it may also play a direct pathophysiological effector role. Use of recombinant IL‐18bp or anti‐IL‐18 therapies may therefore be useful for treatment of inflammation in WAS, and further studies of larger cohorts evaluating IL‐1 family cytokines are warranted.

## Conflict of interest

The authors declare no commercial or financial conflict of interest.

### Peer review

The peer review history for this article is available at https://publons.com/publon/10.1002/eji.202049024.

## Supporting information

Supporting information

## Data Availability

Further data are available from the corresponding author upon reasonable request.
