# Supplementary material for: IL‐18: A potential inflammation biomarker in Wiskott–Aldrich syndrome
Source: Eur J Immunol. 2021 Feb 5;51(5):1285–8. doi: 10.1002/eji.202049024 (PMC8436742; doi:10.1002/eji.202049024)
Supplement: Supplementary file 1 — Supporting information [file EJI-51-1285-s001.pdf]

IL-18: a potential inflammation biomarker in Wiskott Aldrich syndrome
Supporting information

Ethical approval

Informed consent was obtained from the patients and their parents who participated in this study (REC 06/Q0508/16).

Author contributions

ER, MBE, AW and AJT designed the study, drafted and reviewed the manuscript. ER and YH obtained and analysed the data.

Supplemental materials and methods

Serum samples

Patient and healthy adult donor clotted blood samples were centrifuged at 5000rpm for 5 minutes. Serum was collected and stored at -80°C.

Haematopoietic stem cell transplant and gene therapy procedures

Further details of HSCT and GT procedures can be found at [1] and [2] respectively.

Cytokine and chemokine measurement

Multiple cytokine and chemokines were simultaneously quantified from patient and healthy adult donor serum (IL-1β, IL-6, TNF-α, IFN-γ, IP-10 and MCP-1) by electrochemiluminescence immunoassay using Meso Scale Discovery according to manufacturer's instructions. Data were analysed with Discovery Workbench 4.0. IL-18 and IL-18bp were quantified using ELISA (Human IL-18 Instant ELISA, eBioScience and R&D systems respectively) according to manufacturer's instructions.

Statistical analysis

All graphs and statistical analyses were obtained using GraphPad Prism version 5.

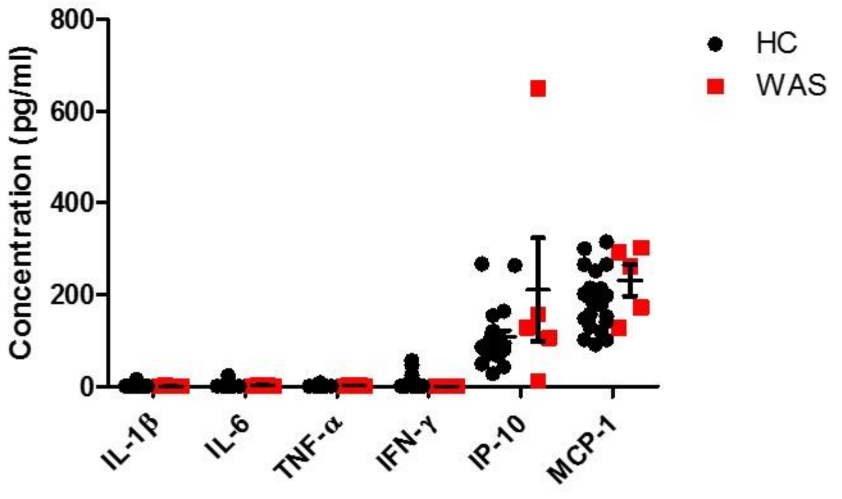

Supplementary Figure 1: Circulating levels of pro-inflammatory cytokines and chemokines in WAS
Serum cytokine and chemokine concentrations were quantified from 19 healthy donors (black dots) and 5 WAS patients (red squares) by multiplex MSD assay. Dots represent mean values of duplicate results and bars represent mean +/- SEM
HC, healthy control; IFN, interferon; IL, interleukin; IP-10, interferon gamma-induced protein 10; MCP-1, monocyte chemotactic protein 1; MSD, mesoscale discovery; SEM, standard error of mean; TNF, tumour necrosis factor; WAS, Wiskott Aldrich syndrome

References

1 **Elfeky, R. A., Furtado-Silva, J. M., Chiesa, R., Rao, K., Amrolia, P., Lucchini, G., Gilmour, K., Adams, S., Bibi, S., Worth, A., Thrasher, A. J., Qasim, W. and Veys, P.,** One hundred percent survival after transplantation of 34 patients with Wiskott-Aldrich syndrome over 20 years. *J Allergy Clin Immunol* 2018.

2 **Hacein-Bey Abina, S., Gaspar, H. B., Blondeau, J., Caccavelli, L., Charrier, S., Buckland, K., Picard, C., Six, E., Himoudi, N., Gilmour, K., McNicol, A. M., Hara, H., Xu-Bayford, J., Rivat, C., Touzot, F., Mavilio, F., Lim, A., Treluyer, J. M., Heritier, S., Lefrere, F., Magalon, J., Pengue-Koyi, I., Honnet, G., Blanche, S., Sherman, E. A., Male, F., Berry, C., Malani, N., Bushman, F. D., Fischer, A., Thrasher, A. J., Galy, A. and Cavazzana, M.,** Outcomes following gene therapy in patients with severe Wiskott-Aldrich syndrome. *JAMA* 2015. **313**: 1550-1563.
